# Assessing neuraxial microstructural changes in a transgenic mouse model of early stage Amyotrophic Lateral Sclerosis by ultra‐high field MRI and diffusion tensor metrics

**DOI:** 10.1002/ame2.12112

**Published:** 2020-04-16

**Authors:** Rodolfo G. Gatto, Carina Weissmann, Manish Amin, Ariel Finkielsztein, Ronen Sumagin, Thomas H. Mareci, Osvaldo D. Uchitel, Richard L. Magin

**Affiliations:** ^1^ Department of Bioengineering University of Illinois at Chicago Chicago IL USA; ^2^ Instituto de Fisiología Biologia Molecular y Neurociencias‐IFIBYNE‐CONICET University of Buenos Aires Buenos Aires Argentina; ^3^ Department of Biochemistry National High Magnetic Field Laboratory University of Florida Gainesville FL USA; ^4^ Department of Pathology School of Medicine Northwestern University Chicago IL USA

**Keywords:** amyotrophic lateral sclerosis, animal models, diffusion tensor imaging, G93A‐SOD1 mice, ultra‐high field MRI

## Abstract

**Objective:**

Cell structural changes are one of the main features observed during the development of amyotrophic lateral sclerosis (ALS). In this work, we propose the use of diffusion tensor imaging (DTI) metrics to assess specific ultrastructural changes in the central nervous system during the early neurodegenerative stages of ALS.

**Methods:**

Ultra‐high field MRI and DTI data at 17.6T were obtained from fixed, excised mouse brains, and spinal cords from ALS (G93A‐SOD1) mice.

**Results:**

Changes in fractional anisotropy (FA) and linear, planar, and spherical anisotropy ratios (C_L_, C_P_, and C_S_, respectively) of the diffusion eigenvalues were measured in white matter (WM) and gray matter (GM) areas associated with early axonal degenerative processes (in both the brain and the spinal cord). Specifically, in WM structures (corpus callosum, corticospinal tract, and spinal cord funiculi) as the disease progressed, FA, C_L_, and C_P_ values decreased, whereas C_S_ values increased. In GM structures (prefrontal cortex, hippocampus, and central spinal cord) FA and C_P_ decreased, whereas the C_L_ and C_S_ values were unchanged or slightly smaller. Histological studies of a fluorescent mice model (YFP, G93A‐SOD1 mouse) corroborated the early alterations in neuronal morphology and axonal connectivity measured by DTI.

**Conclusions:**

Changes in diffusion tensor shape were observed in this animal model at the early, nonsymptomatic stages of ALS. Further studies of C_L_, C_P_, and C_S_ as imaging biomarkers should be undertaken to refine this neuroimaging tool for future clinical use in the detection of the early stages of ALS.

## 1 INTRODUCTION

The introduction of animal models has been one of the major steps forward towards a better understanding of the neuropathological processes occurring in humans. Based on their similarity to the human genome and easy availability, mammalian murine models have been one of the most commonly used representations of neurodegenerative diseases such as Amyotrophic Lateral Sclerosis (ALS).^1^ As such, the phenotypical expression and histological discoveries parallel many of the symptomatology and neuropathological findings observed in patients with ALS.^2^, ^3^ During the last decades, the exponential growth of novel genetic tools has led to the detection of new mutations in the patient population, and thus to help in the development of new transgenic animal models for research and therapeutic discovery purposes.^4^

From the original ALS mouse model representing the familiar form of ALS with the mutation of the superoxide dismutase 1 (G93A‐SOD1) gene,^5^ a growing number of rodent models that express different mutations, such as the fused in sarcoma (FUS),^6^ the C9orf72 hexanucleotide repeat expansion mice,^7^, ^8^ and the transactive response DNA binding protein 43 kDa TDP‐43^9^, ^10^ (among others) have been increasingly developed to address the effects of molecular changes on neuronal degeneration and death. However, such models represent less than 10% of the sporadic cases of ALS. Other models of sporadic ALS mutations, like the wobbler and VPS54 mice,^11^, ^12^ have been used aiming to understand the basic cellular mechanisms of motoneuron diseases, but these abnormalities are likely different from those occurring in ALS.^1^  Despite the growing availability of murine animal models, the G93A‐SOD1 mouse model is the original and longer tested mammalian model of ALS up to date.^13^

In the last decades, the increasing development of MRI systems has been able to allow neuroscientists to analyze in real‐time the neuropathological process occurring in physiological intact biological systems.^14^, ^15^ Also, the advances in computational power, hardware, and gradient strengths has been paramount in high‐field resolution MRI particularly in small animal research.^16^, ^17^, ^18^ Since its inception, diffusion tensor imaging (DTI) has been applied as an imaging technique to evaluate not only microstructure but also the integrity and connectivity of different CNS regions.^19^ Diffusion tensor imaging provides a mathematical model of diffusion anisotropy and is widely used. Parameters, including fractional anisotropy (FA), mean diffusivity (MD), as well as parallel and perpendicular diffusivity, can be derived to provide sensitive, but nonspecific, measures of the altered tissue structure.^20^ The popularity of DTI has been based on its relatively simple computational algorithms based on the directionality of the diffusion tensor's eigenvalues.^21^ Therefore, this diffusion technique is one of the most widely utilized in the scientific and medical field to evaluate axonal injuries across different axonal tracts.^22^, ^23^

The diffusion tensor can be visualized using an ellipsoid where the principal axes correspond to the directions of the eigenvector system.^24^, ^25^ By applying the symmetric properties of this ellipsoid, the diffusion tensor can be decomposed into basic geometric measures to describe the shape of the diffusion tensor models.^26^ In that regard, DTI derived parameters such as linear (C_L_), planar (C_P_), and spherical (C_S_) anisotropy have been only applied to evaluate hippocampal structures on preclinical models of epilepsy.^27^ Here, we extend this approach to characterize such DTI derived morphological parameters in the context of ALS. In previous work, we used ultra‐high field diffusion MRI (UHFD‐MRI) to asses microstructural changes in gray and white matter (GM & WM) applied to different murine models of neurodegenerative diseases.^28^, ^29^ Our results have shown that structural changes can be detected during earlier stages of the disease.^14^, ^30^, ^31^ In line with such studies, we postulate that the intrinsic geometrical properties of monoexponential diffusion signals derived from DTI could add another level of specific information in relation to different neuraxial structures. Hence, in this work we extended our previous investigations on DTI to analyze if parameters representing the DTI tensor contain additional information that: a) determine the specific geometrical characteristics of diffusion tensors across different neuronal tissue interrogated and b) to assess their value as additional biomarkers of disease in the context of neurodegenerative diseases (ALS mice). To test  these hypotheses, we analyzed CNS tissues of an animal model of ALS (G93A‐SOD1 mice) with UHF‐MRI to determine the potential role of derived DTI anisotropic parameters in ALS.

## 2 METHODS

### 2.1 Theory

The tensor field data were diagonalized using the standard analytical methods and eigenvalues were obtained (λ_1_, λ_2_, and λ_3_) to calculate Fractional Anisotropy (FA) as described in Eq. 1. Additionally, the tensor field was used to compute the DTI metrics, such as linear anisotropy (C_L_), planar anisotropy (C_P_), and spherical anisotropy (C_S_) as described in Equation 2, 3, 4, respectively.^32^, ^33^

(1) $$\text{FA}=\sqrt{\frac{1}{2}}\frac{\sqrt{{\left(\lambda_{1}-\lambda_{2}\right)}^{2}+{\left(\lambda_{1}-\lambda_{3}\right)}^{2}+{\left(\lambda_{2}-\lambda_{3}\right)}^{2}}}{\sqrt{\lambda_{1}^{2}+\lambda_{2}^{2}+\lambda_{3}^{2}}}$$

(2) $$C_{L}=\left(\lambda_{1}-\lambda_{2}\right)/\text{trace}$$

(3) $$C_{P}=2\left(\lambda_{2}-\lambda_{3}\right)/\text{trace}$$

(4) $$C_{S}=3\left(\lambda_{3}\right)/\text{trace}$$

where; trace = (λ_2_ + λ_2_ + λ_3_)

**Figure 1:**
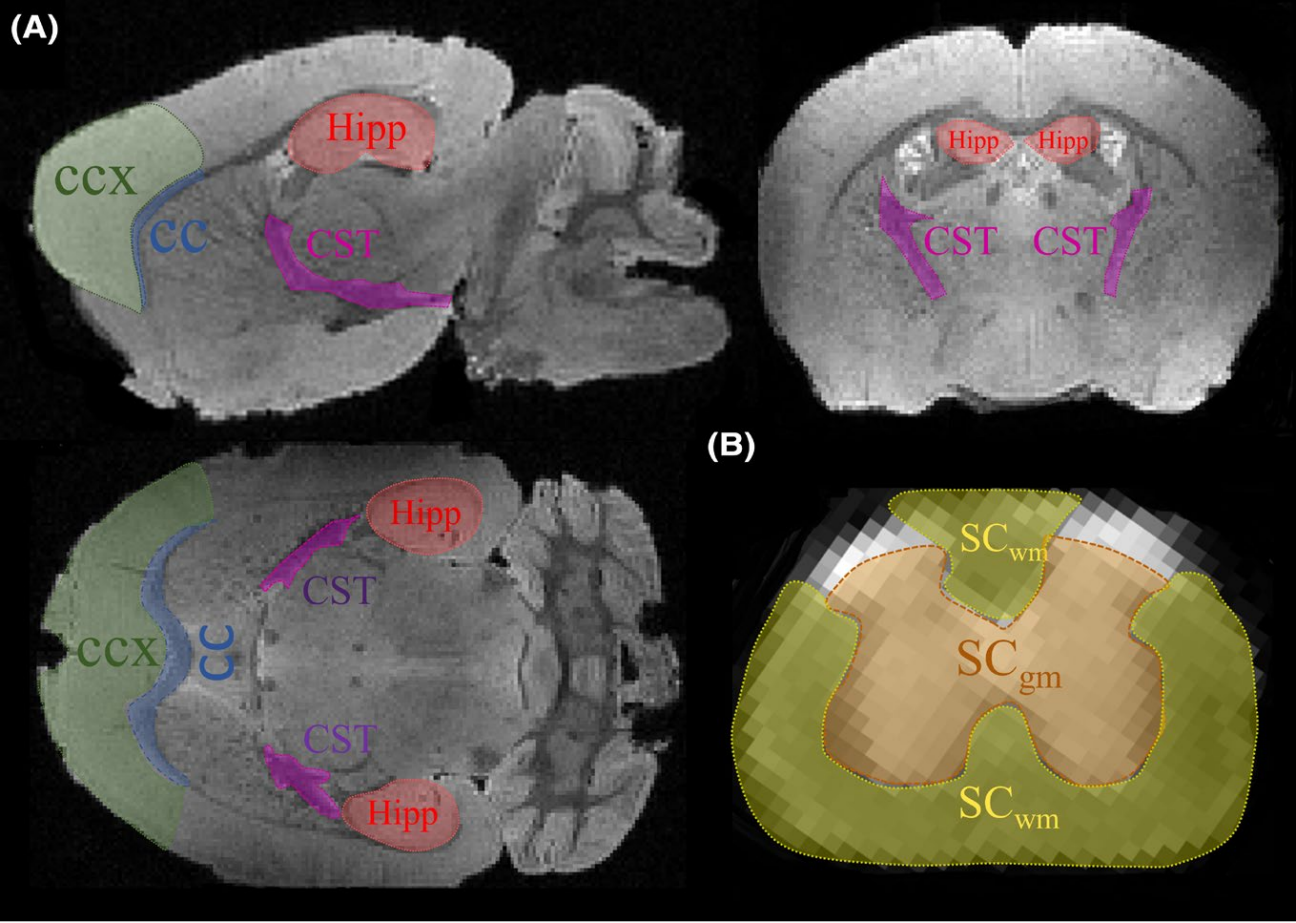
Anatomical maps and areas of segmentation in the ALS mouse. A, T2 map anatomical representation of a mouse brain showing different white (WM) and gray matter (GM) segmented regions used for diffusion tensor imaging (DTI) metrics analysis. B, MRI diffusion map at b0 from a mouse spinal cord (SC). SC segmentation was centered in the lumbar WM and GM regions

FA and DTI metric maps were calculated, and a mean value of each parameter was extracted and calculated from each ROI (Figure 1).

### 2.2 Animals

All procedures used to obtain tissues followed an approved protocol from the animal care committee (ACC) at the University of Illinois at Chicago (UIC). In any situation of animal distress or pain, animals were sacrificed in carbon dioxide using standard protocols For MRI and histology imaging methods, ALS mice were obtained from the Jackson Laboratory (JAX#004435) and bred on a C57BJ6 background, overexpressing the SOD1 transgene with the G93A mutation. The G93A‐SOD1 mice in this background have been previously characterized and they develop motor symptoms at approximately 110 days of age and die around 160 days.^34^, ^35^ We considered three groups of animals for this work: a wild‐type control group (WT), a presymptomatic group at postnatal day 80 (P80) and a symptomatic group at postnatal day 120 (P120). For MRI studies, a total of 12 animals were used: wild‐type (WT) control (n = 4) and ALS (G93A‐SOD1) mice (n = 4 per group). Mice had easy access to food and water and were checked daily to assess their level of well‐being and health. Additional animals WT, P80, and P120 were used for further histological analysis, Specifically, we evaluated morphologic neuronal anomalies in the context of ALS, using additional mouse reporters expressing a yellow fluorescent protein (YFP) transgene specifically associated with a neuronal Thy1 promoter, were chosen. The first reporter group was chosen for the high YFP expression in axons located in spinal cord areas, the so‐called YFP‐J16 mice (JAX#003709). The second group of YFP mice was chosen for its mild fluorescent Thy1 expression and higher background, making it ideal to study individual neuronal structural details in the cerebral cortex and hippocampus, so‐called YFP‐H mice (JAX#003782). Detailed molecular and neuronal population differences between both YFP reporters have been extensively described in previous work.^36^, ^37^

### 2.3 MRI and DTI protocol

Animals were rendered unconscious with CO_2_ inhalation, then transcardially perfused with a PBS and 4% paraformaldehyde (PFA) solution. After the skull was opened, mouse brains were extracted intact and immersed in PFA (>48 hours). Before scanning, brains were soaked overnight in phosphate‐buffered saline (PBS) (Corning cellgro, catalog #21‐040‐CV, lot#12417001) to remove free fixative. Three brains were stacked in a 10 mm inner diameter glass tube (Fisher Scientific, cat#14‐961‐26) and surrounded with fluorocarbon oil (Fluorinert®, 3M, Maplewood, MN). Images from brains were acquired with a 17.6T vertical‐bore Avance II scanner using a 25 mm RF coil. Spinal cords (SC) were placed on a 5 mm NMR tubes (New Era #NE‐MQ5‐7, 300‐400 MHz) and scanned in a 5 mm RF coils as described in previous studies.^38^

Two imaging sessions of six mouse brains (N = 12), with a total of 170 MRI slices were acquired, coronally centered and oriented along the rostral‐caudal axis of each brain. Additionally, a group of six spinal cords was imaged (16 axial slices per SC) using an acquisition centered in the lumbar region. For all the scans, diffusion‐weighted images were acquired using a spin‐echo sequence with TR = 10 000 ms and TE = 20 ms, interleaved 0.2‐mm‐thick slices, field of view = 25 × 25 × 34 mm^3^ in each block of slices, in‐plane acquisition matrix = 125 × 125 × 170, for an isotropic image resolution of 200 μm. A multi‐slice 2D acquisition was used for all the samples. Diffusion‐weighted images were acquired in the cool bore (20°C) of the magnet with b = 0 s/mm^2^ with b = 1000 s/mm^2^ in 20 directions. The acquisition time was approximately 19 hours per session. FSL software was used to calculate each eigenvalue and the anisotropy parameters.^39^ ROIs from each central nervous system structure  were manually segmented following anatomical landmarks described in the standard stereotaxic coordinate mouse brain and spinal cord atlases and data extracted using ITK‐SNAP.^40^

### 2.4 Histology

After MRI scanning, oil media was removed, and brains were placed in increasing concentration of sucrose solutions [5%‐30%] for an additional 24 hours for cryo‐protection. After embedding in optimal cutting temperature (OCT) polymer compound (Tissue Tek, Sakura, Finetek, cat #4583), 50‐µm‐thick brain sections were obtained using a microtome (Leica cryostat CM 1850 Cryostat, Buffalo Grove, IL). Brain sections were mounted on slides (Fisher‐brand Superfrost cat# 12‐550‐15) and dried for 15 minutes. Then, the OCT was removed by washing three times with Tris base buffer (TBS). Slides were dried and mounted in Vecta‐Shield mounting media (Vector Laboratories, Burlingame, CA). Cortical and hippocampal images of the YFP(H) mice reporter group were acquired with a Carl Zeiss structural confocal microscope (ApoTome.2, Oberkochen, Germany). Imaging of the spinal cord was performed on a subset of YFP(J16) mouse brain slices on a confocal Olympus microscope (FluoView FV1000, Shinjuku, Tokyo, Japan). Representative histological sections were selected matching stereotaxic coordinates.^41^, ^42^ Fluorescent confocal microscopy images were obtained with a 534 nm laser channel using standard techniques previously described^29^, ^37^ and images processed using ImageJ software.^43^, ^44^

### 2.5 Statistical analysis

Quantitative data were tabulated and analyzed using statistical GraphPad Prism 6 software (La Jolla, CA). Minimum group size of animals per experimental group was established using power analysis and sample size calculations based on the results from preliminary experiments. For quantitative analysis, one‐way ANOVA and Tukey's post hoc tests were used to determine statistical differences among experimental animal groups. A value of *P* < .05 was used to demonstrate statistical significance. Results were replicated by the application of nonparametric statistical tools (Mann‐Whitney test). Error bars in all the figures represent standard error of the mean (SEM).

## 3 RESULTS

### 3.1 Each neuronal tissue structure in the mice corresponded to a specific DTI metric

**Figure 2:**
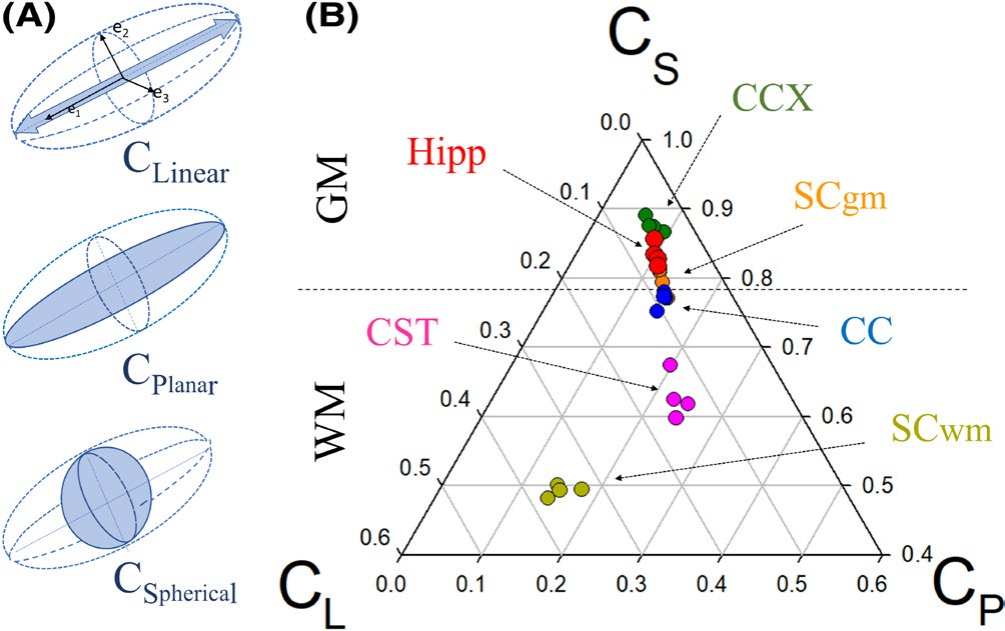
MRI Diffusion tensor derivatives in different tissues of a wild‐type mouse. A, Diagram representing the different parameters used to assess tensors geometry, such as linear, planar, and spherical anisotropy (CL, CP, and CS, respectively) and its spatial relationship with eigenvalues (e). B, Representation of CL, CP, and CS in Bayesian coordinate plots across different central nervous system (CNS) region in a wild‐type rodent (WT). Note that different gray and white matter structures yield tensors with a unique & different geometry. Animals (n = 4), *P < .05, **P < .01. Abbreviations: WM, white matter; GM, gray matter; CC, corpus callosum; CST, corticospinal tract; CCX, cortex; Hipp, hippocampus; SC_wm_, spinal cord white matter; SC_gm_, spinal cord gray matter

**Figure 3:**
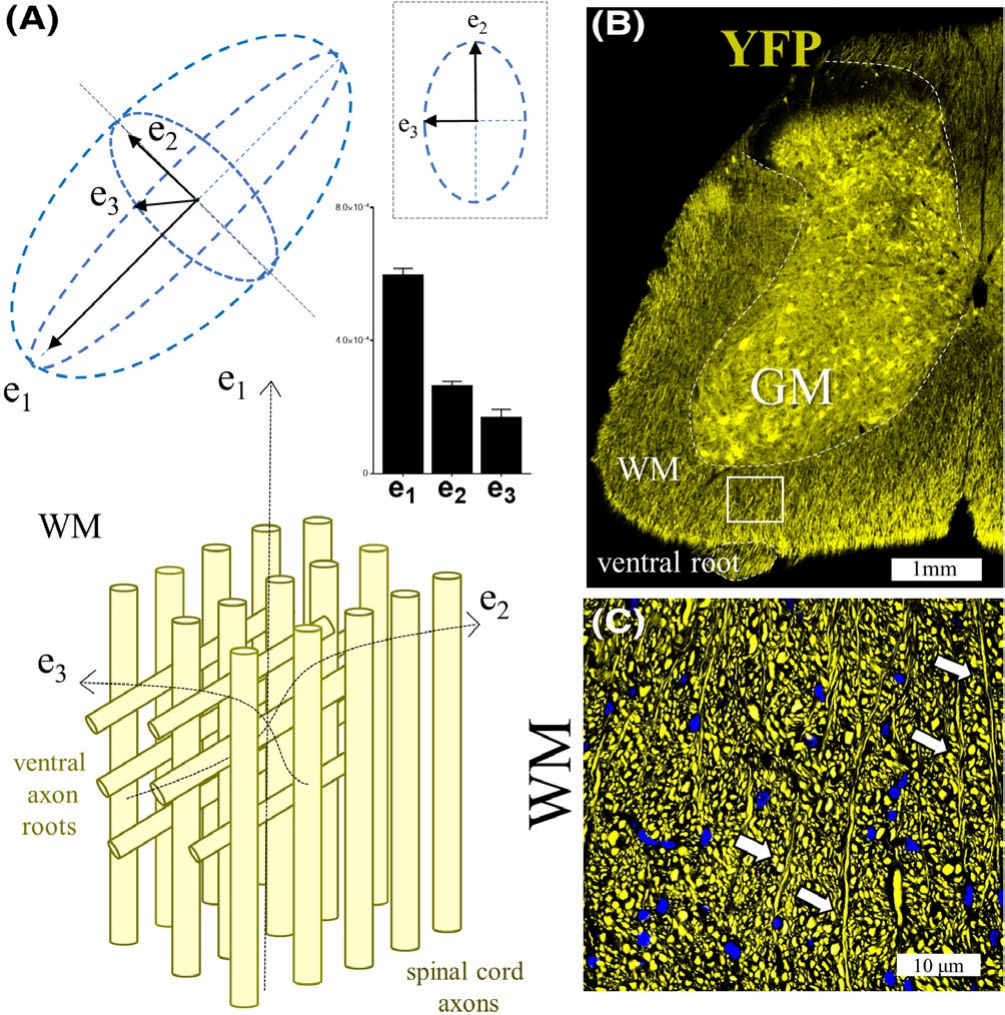
Asymmetry across geometric planes of diffusion tensors are associated to tissue complexity in the spinal cord of the ALS mouse. A, Diffusion tensor diagram showing relative differences in white matter spinal cord (SC) eigenvalues (e) in a naïve mouse. In the perpendicular view of the tensor, note the asymmetry between e2 and e3 (above). Tridimensional complexity and tissue inhomogeneities could be associated to the relative proportion of axonal fibers crossing from the gray matter region exiting on each spinal cord root (diagram below). B, Perpendicular histological section from a lumbar spinal cord region in a YFP axon labeled mouse showing a representative region of crossing fibers. C, Higher magnification of the square area revealed a highly prevalent population of crossing axons (arrows). Abbreviations: YFP, yellow fluorescent protein; WM, white matter; GM, gray matter. Scale bar in Figure 3B = 1 mm. Scale bar in Figure 3C = 10 microns

Initial analyses were centered on three different WM and three GM structures (Figure 1). Following the literature, FA values in GM were significantly lower than in WM, demonstrating a higher degree of complexity and anisotropy in such structures. For example, for GM structures such as the prefrontal cortex (CCX), and the hippocampus (hipp), the values of FA were 0.16 ± 0.004 and 0.18 ± 0.009, respectively, whereas for the spinal cord GM (SC_gm_) the FA was 0.22 ± 0.009. Conversely, for WM structures such as the anterior segment of the corpus callosum (CC), the cortico‐spinal tract (CST), and the WM of the spinal cord (SC_wm_) the FA values were as follows: 0.42 ± 0.013, 0.46 ± 0.006, and 0.63 ± 0.01, respectively (Figure 2). Overall, the C_L_ was greater in WM than GM regions, the C_P_ was slightly greater in WM structures, and the C_S_ was substantially greater in the GM. Interestingly, SC_wm_ had a larger C_L_ and C_S_ values when compared to other WM regions (Table 1). As such, tensor metric differences across each WM tracts can be accounted for their specific eigenvalues, marking the proportion of crossing fibers on each WM structure (Figure 3).

**Table 1:** Summary of DTI Derivative Results in CNS structures of Preclinical Animal Models of Neurodegenerative Diseases

		Control (WT)	Presymptomatic (P80)	Symptomatic (P120)
Cortex (Prefrontal)	FA	0.160 ± 0.004	0.140 ± 0.002 (↓13%)*	0.120 ± 0.006 (↓25%)**
	C_L_	0.050 ± 0.003	0.050 ± 0.001	0.050 ± 0.002
	C_P_	0.090 ± 0.004	0.070 ± 0.002 (↓22%)*	0.070 ± 0.001 (↓22%)**
	C_S_	0.860 ± 0.001	0.870 ± 0.003	0.880 ± 0.003
Hippocampus	FA	0.180 ± 0.009	0.150 ± 0.010 (↓16%)*	0.130 ± 0.007 (↓27%)**
	C_L_	0.060 ± 0.003	0.060 ± 0.005	0.050 ± 0.006 (↓16%)*
	C_P_	0.100 ± 0.005	0.080 ± 0.002 (↓20%)*	0.070 ± 0.005 (↓30%)**
	C_S_	0.830 ± 0.008	0.860 ± 0.011	0.880 ± 0.002
Spinal Cord (GM)	FA	0.220 ± 0.009	0.200 ± 0.004	0.180 ± 0.007 (↓21%)**
	C_L_	0.080 ± 0.002	0.070 ± 0.003 (↓13%)*	0.060 ± 0.005 (↓25%)**
	C_P_	0.140 ± 0.007	0.120 ± 0.002 (↓14%)*	0.110 ± 0.006 (↓ 21%)**
	C_S_	0.790 ± 0.008	0.810 ±0.004	0.830 ± 0.008
Corpus Callosum (Genu & Body)	FA	0.420 ± 0.013	0.330 ± 0.017 (↓21%)**	0.300 ± 0.017 (↓29%)**
	C_L_	0.200 ± 0.004	0.140 ± 0.009 (↓30%)**	0.120 ± 0.006 (↓40%)**
	C_P_	0.160 ± 0.003	0.140 ± 0.006 (↓13%)*	0.130 ± 0.005 (↓18%)*
	C_S_	0.650 ± 0.004	0.720 ± 0.014 (↑11%)*	0.740 ± 0.010 (↑14%)*
Cortico‐Spinal Tract (CST)	FA	0.460 ± 0.006	0.410 ± 0.010 (↓11%)*	0.370 ± 0.012 (↓20%)**
	C_L_	0.230 ± 0.011	0.200 ± 0.008 (↓13%)*	0.180 ± 0.013 (↓22%)**
	C_P_	0.150 ± 0.007	0.130 ± 0.004 (↓13%)*	0.110 ± 0.007 (↓27%)**
	C_S_	0.620 ± 0.018	0.680 ±0.005 (↑10%)*	0.710 ± 0.015 (↑15%)*
Spinal Cord (WM)	FA	0.640 ± 0.011	0.560 ± 0.06 (↓13%)*	0.510 ± 0.025 (↓20%)**
	C_L_	0.410 ± 0.006	0.350 ± 0.010 (↓15%)*	0.290 ± 0.019 (↓29%)**
	C_P_	0.150 ± 0.003	0.150 ± 0.008	0.140 ± 0.006
	C_S_	0.440 ± 0.004	0.490 ± 0.004 (↑ 11%)*	0.560 ± 0.024 (↑ 27%)**

Note

Values as mean ± SEM.

Abbreviations: ↑%, percentage increase from wild‐type; ↓%, percentage decrease from WT; ALS, amyotrophic lateral sclerosis; C_L_, linear anisotropy; CNS, central nervous system; C_P_, planar anisotropy; C_S_, spherical anisotropy; FA, fractional anisotropy; WT, wild‐type.

* *P* < .05 from control;

** *P* < .01 from control.

### 3.2 Progressive and distinct changes in DTI metrics can be observed across different neuraxial regions of the ALS mice

**Figure 4:**
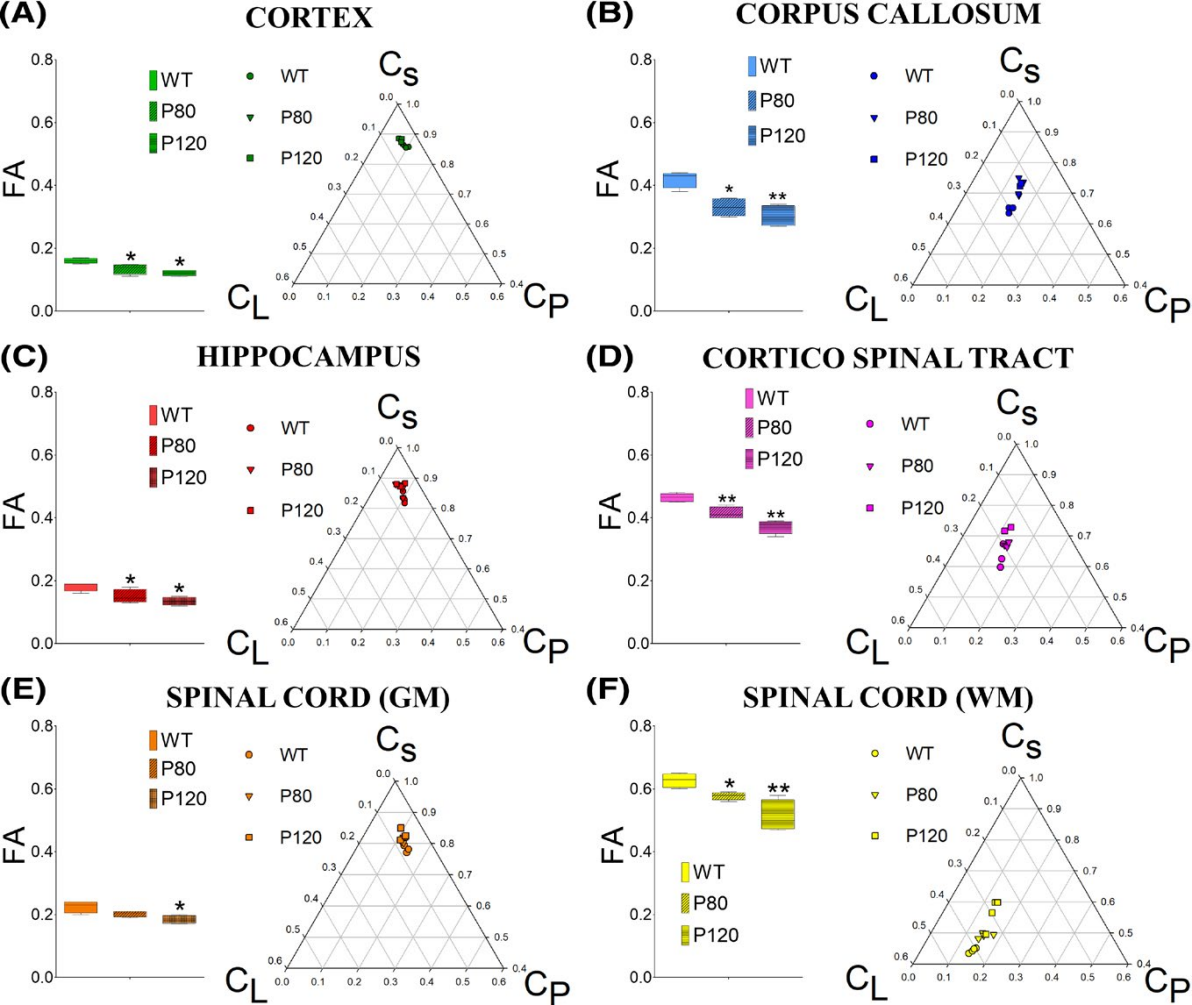
Longitudinal changes in tensor derived geometry across different white and gray matter neuraxial tissues (wild‐type vs presymptomatic and symptomatic G93A‐SOD1 mouse). A,C,E, Representation of fractional anisotropy (FA) and tensor geometries measured in cerebral superficial gray matter (GM) regions in the prefrontal cortex (CCX) (A), deep GM regions, such as the hippocampus (C) as well as the GM portion of the spinal cord (SC_gm_) (E) captured by linear, planar, and circular anisotropy (CL, CP, and CS, respectively). B, D, F, Analysis of tensor metrics changes in cerebral white matter (WM) regions, such as the corpus callosum (CC) (B) and the corticospinal tract (CST) (D) as well as in the WM region of the lumbar spinal cords (SC_wm_) (F). Note the relatively small change in tensor shape between the control (WT) group and the presymptomatic (P80) and symptomatic (P120) animal groups in GM, vs the wider changes in WM structures. Animals (n = 4), *P < .05, **P < .01. Abbreviations: WM, white matter; GM, gray matter; CC, corpus callosum; CST, corticospinal tract; CCX, cortex; Hipp, hippocampus; SC_wm_, spinal cord white matter; SC_gm_, spinal cord gray matter

**Figure 5:**
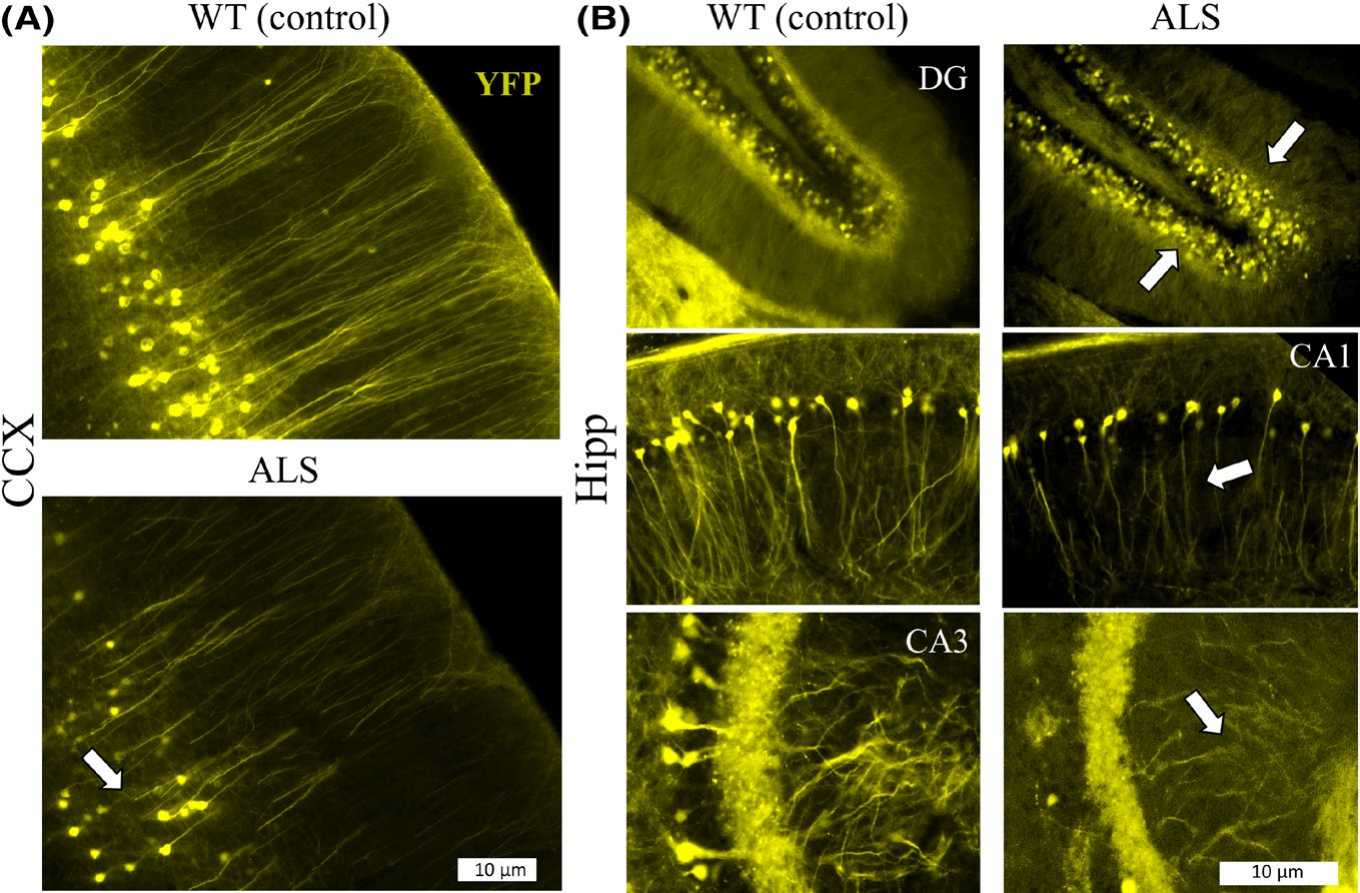
Early structural anomalies in neuronal connectivity can be detected in the cortical gray matter of the YFP‐G93A‐SOD1 mouse*.* A, Yellow Fluorescent Protein (YFP) expression in neurons of the prefrontal cortex showed a significant impairment in neuronal architecture in the ALS mice (arrow). B, YFP expression in different segments of the hippocampus, such as the dental gyrus (DG), cornus Ammonis 1 (CA1), and cornus Ammonis 3 (CA3) showed early structural anomalies in dendritic and axonal structures (arrows) in the ALS mouse. Abbreviations: ALS, Amyotrophic Lateral Sclerosis; Hipp, Hippocampus; DG, dental Gyrus; CA1, Cornus Amonus segment 1; CA3, Cornus Amonus segment 3; CCX, cortical region; Hipp, hippocampus. Scale bar in Figure 5A, B = 10 microns

As the disease progressed, DTI derived parameters changed accordingly. Overall, greater decreases in C_L_ was observed in WM structures at presymptomatic (P80) and symptomatic stages (P120) in the anterior segment of the CC: (WT C_L_ = 0.200 ± 0.004) vs (P80 C_L_ = 0.140 ± 0.009, *P* < .01) vs (P120 C_L_ = 0.120 ± 0.006, *P* < .01), in the CST: (WT C_L_ = 0.230 ± 0.011) vs (P80 C_L_ = 0.200 ± 0.008, *P* < .05) vs (P120 C_L_ = 0.180 ± 0.010, *P* < .01), as well as in the SC_wm_: (WT C_L_ = 0.410 ± 0.006) vs (P80 C_L_ = 0.350 ± 0.010, *P* < .05) vs (P120 C_L_ = 0.290 ± 0.019, *P* < .01). Although relatively larger C_P_ changes were seen in GM structures such as the CCX and Hipp, greater changes in C_S_ were seen in all the WM structures studied, such as the CC: (WT C_s_ = 0.650 ± 0.004) vs (P80 C_s_ = 0.720 ± 0.014, *P* < .05) vs (P120 C_s_ = 0.740 ± 0.010, *P* < .05), and the CST: (WT C_s_ = 0.620 ± 0.018) vs (P80 C_s_ = 0.680 ± 0.005, *P* < .05) vs (P120 C_s_ = 0.710 ± 0.015, *P* < .05); with broader changes found in the SC_wm_ regions (WT C_s_ = 0.440 ± 0.004) vs (P80 0.490 ± 0.004, *P* < .05) vs (P120 C_s_ = 0.560 ± 0.024, *P* < .001) (Table 1). Overall, the changes detected for DTI‐derived parameters were more prominent in WM compared with GM structures (Figure 4). Ground truth underlying these MRI results can be seen in the microstructural changes observed in histological preparations of GM (Figure 5).

### 3.3 Assessing differential structural diffusion tensor changes in specific spinal cord regions of the ALS mice

**Figure 6:**
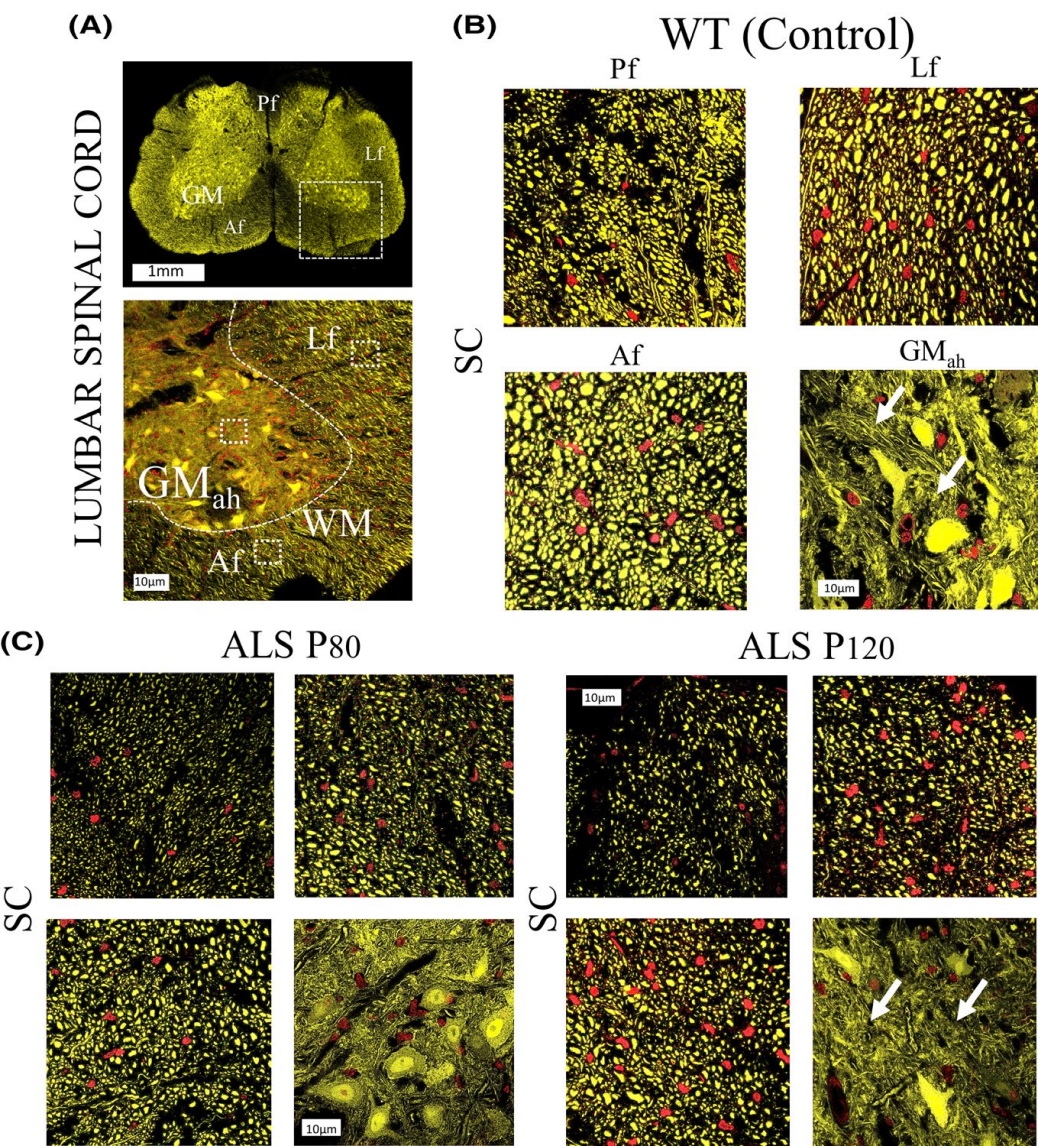
Structural fluorescent changes in different gray and white matter regions of the ALS mouse spinal cord. A, Lumbar spinal cord (SC) cross section of an ALS transgenic mouse labeled with yellow fluorescent protein (upper figure). Anterior area of the SC area at higher magnification in the anterior region (lower figure). Higher magnification of white matter and gray matter regions of interest (ROIs) are marked (squared dots). B, Fluorescent microscopic imaging of different WM ROIs, such as the anterior funiculi (Af), lateral funiculi (LF), and posterior funiculi (Pf) and additional optical imaging of the gray matter anterior horn (GM_ah_). C, Fluorescent comparative images from ALS mouse at presymptomatic (P80) (right side) and symptomatic (P120) stages (left side). Note the axonal degeneration pattern in different WM ROIs and the increase in neuropil entanglement and tissue complexity in the GM_ah_ region as disease progresses (arrows). Nuclear counterstaining with propidium iodine (red). Scale bar in Figure 6A = 1 mm. Scale bar in Figure 6B, C = 10 microns. Abbreviations: WM, white matter; GM, gray matter. Af, anterior funiculi; Lf, lateral funiculi, Pf, posterior funiculi

**Figure 7:**
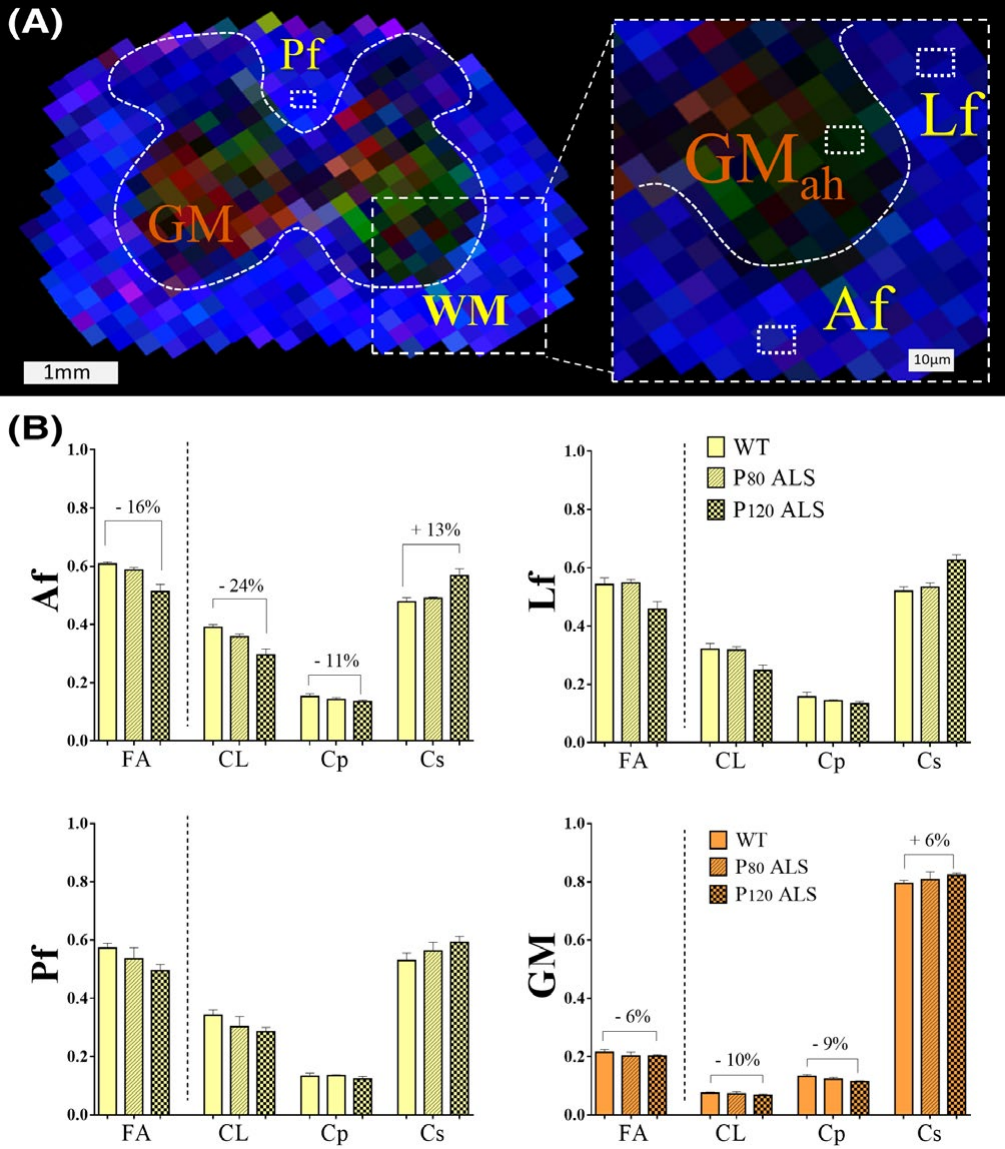
Changes in DTI metrics across different regions of spinal cord in the ALS mice. A, Lumbar FA map of the ALS mice showing the segmentations from different WM ROIs, such as the anterior funiculi (Af), lateral funiculi (Lf), and posterior funiculi (Pf) and gray matter (GM) anterior horn (GM_ah_) areas matching previous histological regions in Figure 6. Scale bar = 1 cm. B, Analysis of DTI derivatives from WM and GM showed increased differences between WT and ALS mice compared in GM region indicating that  Gaussian DTI metrics can better capture differences in WM between control and ALS mice than changes in GM. Scale bar = 10 microns. Animals (n = 4), *P < .05, **P < .01. Abbreviations: WM, white matter; GM, gray matter. Af, anterior funiculi; Lf, lateral funiculi, Pf, posterior funiculi

Expanding previous animal ALS studies,^29^ we used UHF‐MRI to describe the histological structural changes occurring in the spinal cord of the ALS mouse (Figure 6). We analyzed specific WM ROIs of the lumbar spinal cord, such as the anterior funiculi (Af), lateral fasciculi (Lf), and posterior fasciculi (PF) as well as the gray matter anterior horn (GM_ah_). Changes in DTI derivatives across different funiculi (WT vs P120 ALS mice) demonstrated higher variations compared to SC GM. As an example, in the Af region variables were as follows: (WT C_L_ = 0.390 ± 0.009) vs (P120 C_L_ = 0.296 ± 0.020, 16% decrease), (WT C_p_ = 0.152 ± 0.009) vs (P120 C_p_ = 0.135 ± 0.005, 11% decrease), and (WT C_s_ = 0.477 ± 0.013) vs (P120 C_s_ = 0.567 ± 0.023, 13% increase). As such, lower variation was seen in GM_ah_; (WT C_L_ = 0.075 ± 0.002) vs (P120 C_L_ = 0.067 ± 0.004, 6% decrease), (WT C_p_ = 0.132 ± 0.006) vs (P120 C_p_ = 0.115 ± 0.002, 9% decrease), and (WT C_s_ = 0.792 ± 0.008) vs (P120 C_s_ = 0.822 ± 0.007, 6% increase) (Figure 7).

## 4 DISCUSSION

The use of transgenic animal models imposes a significant advantage in neurobiology in the detailed analysis of biomarkers.^45^ Despite the recent introduction of additional animal models of ALS,^46^, ^47^ the vast majority of preclinical mammalian studies are based on rodents.^48^ The possibility of neuronal labeling fusing specific fluorescent tags has allowed neuroscientists to use the tagged fusion expressed in the animals not only to improve the visualization of axonal degeneration in diverse experimental conditions but also as a ground truth to enhance our understanding and characterization of MRI diffusion signals.^49^, ^50^, ^51^ In recent years, DTI metrics have been used in several  neuro‐oncological,^52^, ^53^, ^54^, ^55^ as well as neuroinfectious^56^, ^57^, ^58^, ^59^ clinical scenarios (Table 2). In oncological conditions, a decrease in C_P_ and C_L_ was associated with an increase in C_S_, probably due to a significant increase in tissue cellularity. In neurological and extra neurological^60^ inflammatory pathologies, the C_P_ and C_L_ component increased with a correlative drop in the C_S_ component, possibly related to the increase in edema and the redistribution of the intra and extracellular components, similar to tensor metric measurements in chronic brain edema scenarios due to metabolic pathologies (liver failure).^61^, ^62^ Although DTI metrics have been a useful imaging marker to neuromonitoring various clinical neuropathological processes, the underlying microstructural changes have not been fully explored, particularly in the context of basic neurodegenerative diseases research. During its progression, neurodegenerative diseases combine several complex elements of neuroinflammation and microstructural remodeling across different cellular compartments that are fairly documented in vitro,^63^, ^64^, ^65^, ^66^, ^67^, ^68^ as well as in vivo^14^, ^30^, ^69^, ^70^ experimental models, but still require further characterization. Thus, this is the first study to apply DTI metrics to investigate the effects of neurodegenerative disease in an ALS transgenic murine model.

**Table 2:** Previous applications of diffusion tensor imaging derivatives across different pathological conditions

Study design	Pathology & tissue structure	FA	C_L_	C_P_	C_S_	Citation
Rodent model (Pilocarpine & Kainic acid induced)	Epilepsy [Hippocampus DG]	Increased	Increased	Increased	Decreased	Salo et al^27^
Clinical	Brain tumor [Low vs High grade Glioma]	Decreased	Decreased	Decreased	Increased	El‐Serougy et al^52^
Clinical	Brain tumor [Glioblastoma]	Decreased	Decreased	Decreased	Increased	Cortez‐Conradis et al^53^
Clinical	Brain tumor [WM tracts around Glioblastoma]	Decreased	Decreased	Decreased & NS changes	Increased	Mormina et al^55^
Clinical	Brain tumor [Glioblastoma]	Decrease	Decreased	NS changes	Increase	Roldan‐Valadez et al^54^
Clinical	Neurocysticercosis [Vesicular to Calcified stage]	Increased	Increased	Increased	Decreased	Gupta et al^58^
Clinical	Brain abscess [Hemorrhagic vs Nonhemorrhagic wall]	Increased	Increased	—	Decreased	Gupta et al^89^
Clinical	Brain tumor [High vs Low grade Infiltrating Astrocytoma]	Increased	—	—	Decreased	Jolapara et al^90^
Clinical	Brain tumor [Glioblastoma]	Decreased	Decreased	Decreased	Increased	Saksena S et al^91^
Clinical	Brain tumor [Atypical vs Benign Meningiomas]	Increased	Increased	Increased	Decreased	Jolapara et al^92^
Clinical	Brain abscess [Wall vs Cavity]	Increased	Increased	Increased	Decreased	Gupta et al^56^
Clinical	Epidermoid Cyst	Decreased	Decreased	Decreased	—	Santosh et al^93^
Clinical	Epidermoid cyst [Lesion vs WM]	Decreased	Decreased	Increased	Increased	Jolapara et al^94^
Clinical	Synovial inflammation [Synovial tissue]	Increased	Increased	Increased	Decreased	Agarwal et al^60^
Clinical	Brain tuberculoma	Increased	Increased	Increased	Decreased	Gupta et al^59^
Clinical	Brain tumor [Low‐grade Gliomas after Radiotherapy] (WM)	Decreased	Decreased	Decrease	Increased	Haris et al^95^
Clinical	Acute on chronic LF [Cerebral Brain Edema] (GM & FWM regions)	FWM (Decreased) CP GM (NS)	IC (Decreased) CP GM (NS)	FWM (NS) CP GM (NS)	FWM (Increased) CP (NS)	Nath et al^61^
Clinical	Acute LF [Brain Edema]	Decreased	Decreased	Decrease	Increased	Rai et al^62^

Note

Abbreviations: (—) no data; C_L_, linear anisotropy; C_P_, Caudate putamen; C_P_, planar anisotropy; C_S_, spherical anisotropy; WM, White matter; DG, Dental Gyrus; FA, fractional anisotropy; FWM, frontal White matter; GM, Gray matter; IC, internal capsule; LF, liver failure; NS, nonsignificant.

In this study, we analyzed the structural anomalies from the imaging & tensor shape perspective. The findings presented here aim to advance our current understanding of neurodegenerative disease in experimental models by providing additional information about the early pathological and microstructural changes in an ALS mouse model. Our results from our preclinical model indicated an overall longitudinal reduction in C_P_ and C_L_ as well as an increase C_S_ (contrary to what would be expected) due to a decrease in cellular elements during the neurodegenerative process and resembling the neurooncological hyperproliferative clinical scenarios described before. Nevertheless, even though an initial increase in cellular elements could occur as a consequence of the neuroinflammatory and infiltrative processes well documented in the literature^71^, ^72^, ^73^, ^74^, ^75^; it is also possible that a relative reduction of the neuraxial volume could determine a relative increase in cellular density as described in our previous histological finding.^29^, ^38^

Previous DTI work in canine animal models determined that the distribution of data for the WM internal capsule differed markedly from the WM centrum semiovale region.^33^ Furthermore, data for the internal capsule were distributed in a relatively tight cluster, possibly reflecting the compact and parallel nature of its fibers, whereas data for the centrum semiovale were more widely distributed, consistent with the less compact and often crossing pattern of its fibers. This indicates that the tensor shape plot technique can depict data in similar WM regions as being alike, adding more specificity to these parameters. Results presented in our studies (Table 2) have shown a clear difference in tensor geometry between WM (CC, CST, and SC_wm_) and GM (CCX, Hipp, and SC_gm_) neuraxial structures. A careful look at each tensor shape has shown significant differences within cranial (CC and CST) and extracranial (SC_wm_) fiber structures (Figure 2). Thus, it is possible that such differences can be biologically explained by the fiber's content and the proportion of different fiber orientations, imprinting an asymmetry in the eigenvalues and further relation with the tensor shape, as corroborated by fluorescent microscopy evaluations (Figure 3) and previous studies.^76^ For instance, The Cs values across different WM tracts in WT animals indeed show differences that could reaffirm our idea of diffusion tensor asymmetries based on the number of crossing fiber populations (Figure 3A). As the populations of fibers in SCwm as well in CST are mostly formed by fibers leaning in one axis with limited contribution from additional cortical fibers (CST)^77^ or root fibers (SCwm)^76^ (Figure 3B,C). Alternatively, higher CS values from CC regions could be explained due to a significant amount of diverse axonal layers from fibers oriented orthogonal or in oblique directions as observed in other neurodegenerative fluorescent studies.^36^

During the disease progression, we observed a significant variation in diffusion tensor shapes across different structural regions primarily affected in ALS (Figure 4). Particularly, our studies on the relative changes in tensor metrics detected broader geometrical differences in WM than in GM structures. These findings contradict a growing number of reports focused on alterations due to cortical and subcortical remodeling as part of complex ultrastructural dendritic changes occurring in the earlier stages of ALS^78^, ^79^, ^80^, ^81^ linked to behavioral alterations and frontotemporal dementia.^37^, ^82^ These findings were corroborated by microstructural changes in cortical and hippocampal subregions by our YFP‐G93A‐SOD1 histological preparations (Figure 5).

**Figure 8:**
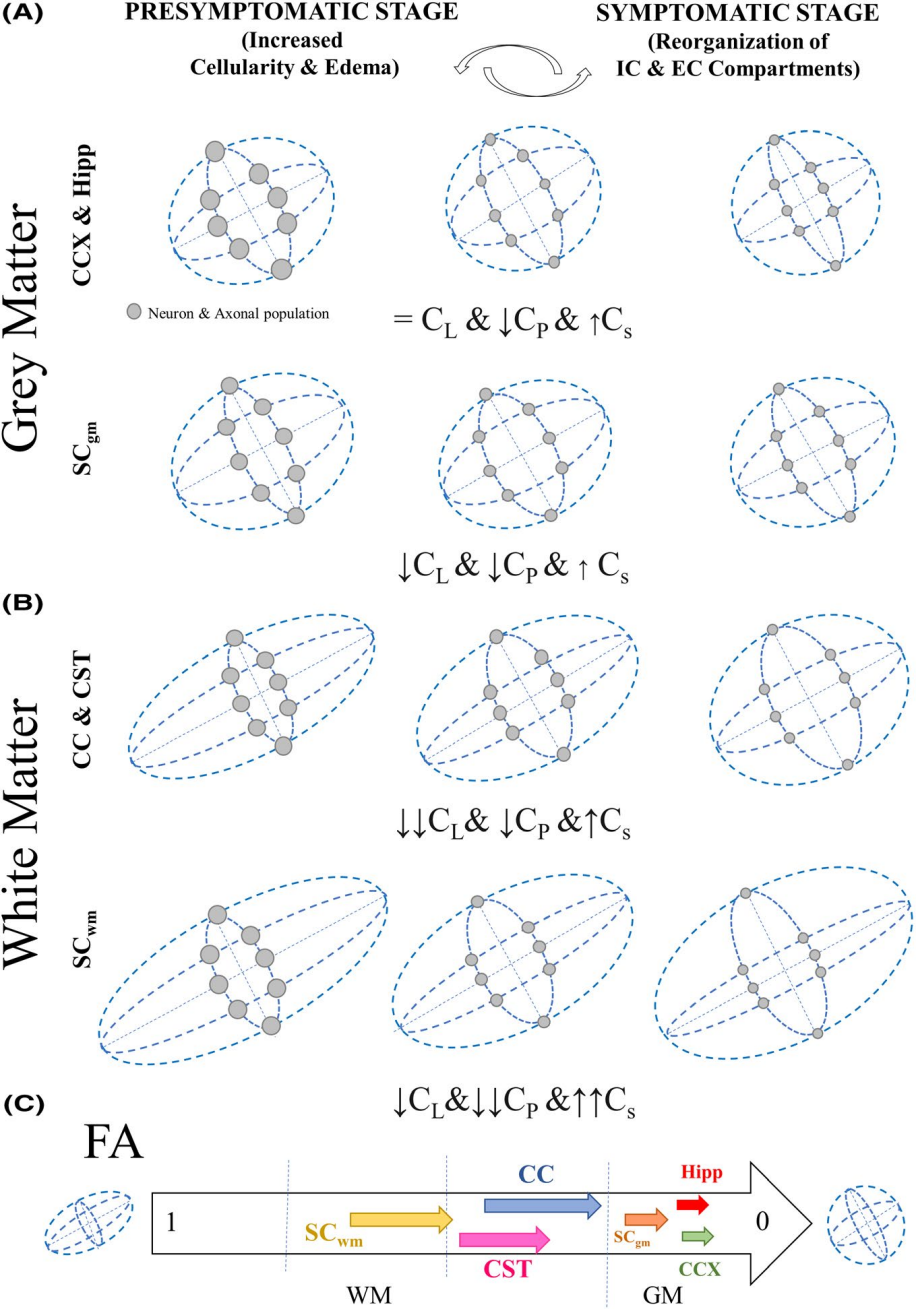
Diagram showing specific geometric changes of diffusion tensors among different neuraxial structures in the ALS mice. A, Changes in gray matter (GM) regions: prefrontal cortex, hippocampus and spinal cord GM. B, Changes in C_L_, C_P_, and C_S_ metrics across different white matter (WM); corpus callosum, cortico‐striatal tract, and spinal cord. Changes in WM and GM are probably associated to specific neuropathological alterations in cellular shape, probably due to neuroinflamatory processes as well as remodeling of intracellular and extracellular compartments. C, Diagram showing progressive changes in tissue organization due to ALS measured by fractional anisotropy (FA) in different WM and GM structures. Abbreviations: IC, intracellular compartment; EC, extracellular compartment; CC, corpus callosum; CST, corticospinal tract; CCX, cortex; Hipp, hippocampus; STR, striatum; SC_wm_ spinal cord white matter. SC_gm_ spinal cord gray matter; WM, white matter; GM, gray matter

Further fluorescent microscopy analysis targeting SC subregions showed that the degeneration process across WM subregions was not equally distributed, demonstrating an early and selective axonal pathology and subsequent disconnection (Figure 6). Such disparity can also be appreciated in the anterior region of the ALS mouse brains, as shown in our original studies. As such, (particularly at the lumbar SC regions) specific changes across different fasciculi have shown earlier and broader DTI geometry changes, compared to SCgm as the disease progresses (Figure 7). In a practical approach, this finding could lead to the documentation of unique brain tissue identifiers leading to an increased optimization of bioimaging markers susceptible to the disease (Figure 8).  Despite we detected a high proportion of neuro‐axonal content  in the areas interrogated, additional neuroinflammatory process should be considered as a factor for tissue alterations.^63^, ^71^ In consequence, the combination of diverse neurodegenerative mechanisms in susceptible neuronal populations could determine the microstructural remodeling process occurring during the development of the disease, and influence the specific tensor geometries observed in the ALS mouse.

The validity of animal models for the study of human diseases such as ALS has been often criticized particularly due to their high phenotypical dependence on their genetic background.^83^, ^84^ From the technical standpoint of MRI diffusion techniques, the biological validity of the results have been often undermined due to the significant resolution mismatch (voxel vs cell sizes), leading to a structural oversimplification of the neurobiological structures representations. Despite the exceptional quality of the UHD‐MRI data, none of the methods demonstrated high anatomical accuracy, which is highly dependent upon parameters of the tractography algorithm. These inaccuracies (highly dependent on acquisition protocol differences) are particularly predominant in scenarios such as long‐range anatomical projections (based on voxel averaged estimates of local fiber orientation) as well as in WM regions of multiple crossing fibers.^85^, ^86^ From our dataset, significant changes in FA (parameter related to tissue organization) were not necessarily followed by changes in DTI metrics, indicating the limitations of these biomarker to describe complex changes in highly heterogenous media. Also, SOD pathology includes not only axonal degeneration, but there can also be other changes such as structural and cellular changes including demyelination, microglial activation among others, which would be more pronounced at the later stage.^38^, ^87^ As such, different neuropathological changes can contribute to diffusion anisotropy changes in other neurodegenerative diseases. In that regard, our future studies will focus on these diffusion derivative parameters in the context of other experimental animal models of neurodegenerative diseases. Lastly, the interrogation of alterations observed in complex brain microstructures (superficial and deep GM regions) could also be limited by the exclusive use of Gaussian diffusion models. Thus, additional implementation of multi‐exponential diffusion models^28^, ^88^ will be required for more accurate representation to improve monitoring, as well as testing new therapeutic strategies in ALS.

## 5 CONCLUSIONS

Anisotropic components of DTI can be used to extract specific microstructural information in a murine model of ALS. Clear differences were observed across different WM neuraxial structures. However, the information obtained on the progression of the disease in this ALS model was limited in the case of GM. Exploring these biomarkers further using anomalous diffusion techniques could represent a step forward to understanding the neuropathological processes linked to ALS and provide a new tool to detect early markers and monitor potential treatments.

## CONFLICT OF INTEREST

None.

## AUTHOR CONTRIBUTIONS

RG and RM designed the MRI experiments. RG, CW, and MA collected the data, conducted the experiments, and analyzed the MRI and histological data. AF collected additional histological data. RG wrote the manuscript. CW, AF, and RM help editing the paper. RS, OU, TM, and RM added their opinions, developed as well as validated the manuscript.
